# The role of emotions in educational processes: the conceptions of teacher educators

**DOI:** 10.3389/fpsyg.2023.1145294

**Published:** 2023-06-22

**Authors:** Rodolfo Bachler, Pablo Segovia-Lagos, Camila Porras

**Affiliations:** ^1^Escuela de Psicología, Facultad de Ciencias Sociales y Artes, Universidad Mayor, Santiago de Chile, Chile; ^2^Colegio San Sebastián de Los Andes, Los Andes, Chile

**Keywords:** learning, beliefs, conceptions, teacher educators, initial teacher training, teaching, emotions, student teachers

## Abstract

The research shows that a very important part of initial teacher education is to reformulate the beliefs that student teachers bring with them from their school experience. These beliefs, which are intuitive in nature, deal with different educational topics and one area that is currently of great importance, due to the emotional turn that the educational system is experiencing, are the beliefs that student teachers hold about the role of emotions in educational processes. In a world full of views that portray emotions as discrete states that are separate from cognitive processes, it is a priority for initial teacher development to train future teachers to hold conceptions that consider the deep emotional-cognitive integration that exists in the human brain. At the same time, this process requires teacher educators (hereafter referred to as TEs) who hold conceptions on this topic that are aligned with the most current scientific knowledge on the subject. However, we do not know how the conceptions that TEs maintain on this topic are, since, until now, research on conceptions has focused on other types of educational topics. Considering the foregoing, this study aimed to evaluate the conceptions that TEs have on this topic, using a questionnaire of dilemmas that was applied to 68 TEs from different universities. The results obtained show that the TEs maintain perspectives on the role of emotions in the teaching and learning processes that oscillate between dualism and emotional-cognitive integration. In addition, it was found that TEs’ perspectives tend to be more integrative when considering attitudinal learning than when considering verbal learning. Finally, the study shows that maintaining integrative perspectives is more difficult when educational situations involve emotions of positive valence that may constitute an obstacle for teaching and learning. The results are discussed, and a series of reflections are elaborated in order to analyze to what extent the beliefs held by TEs are adequate as a cognitive basis for reformulating the conceptions held by student teachers on this issue.

## Justification

Teacher training begins, informally, when future teachers enter the school system as students (Alliaud, 2007). This is what Lortie (1975) called “the apprenticeship of observation,” namely, the problem of having had a series of observational learning about teaching over a long period of time. According to Darling-Hammond (2006), this problem is one of the three main dilemmas that arise during the process of learning to teach. Because of this experience, a long and sustained process of building up beliefs or conceptions^1^ about education begins, which can be considered as an important part of the process of becoming a teacher. As educational research has shown, these beliefs play a role in the construction of teacher identity (García and Sebastián, 2011; Zembylas, 2018) and are closely related to the pedagogical practices that future teachers will develop (Ho et al., 2001; Bueh and Beck, 2015). For these reasons, the study of beliefs constitutes a prominent field of educational research that has achieved great development in recent years (Fortoul Olivier, 2008; Cárcamo and Castro, 2015; Fives and Gill, 2015). What are the beliefs about learning? (Pozo et al., 2006); how teachers conceive the intelligence of students? (Dweck, 1999); and what do they think about the role of emotions in educational processes? (Bächler Silva and Pozo, 2020). These are just a few examples of the wide range of topics addressed from the conceptual and methodological umbrella constituted by the study of teaching beliefs.

### Implicit conceptions and initial teacher training

We are talking about conceptions that are acquired in a nonconscious way, this is the reason why some people refer to them as “implicit conceptions” (Pozo, 2008). On the other hand, as these conceptions do not have a symbolic or linguistic character and are distinguished, rather by their strong corporal nature, they are called “embodied representations” (Gibbs, 2006). These are sensations and images that have been stored in the procedural memory of student teachers, mainly because of their experience as students in the school system. In that place, observing the behavior of their teachers (and experiencing sometimes its negative consequences), they acquire a set of implicit beliefs about educational processes, beliefs that will later take part in the formal process of becoming teachers. In this way, once they are officially incorporated into the initial teacher training (hereafter referred to as ITT), future teachers will bring with them these conceptions, which will interact with the learning contents and educational processes that are developed there. This process will occur regardless of whether a particular training institution considers or not an explicit and specific design for this interaction, since it finally depends on the intrinsic characteristics of learning, a process that is always the result of this relation between the old and the new (Rodrigo et al., 1993, p.245; Catalán, 2011). Therefore, it is not correct to affirm that ITT starts from zero at the university, because, the prior educational background of student teachers’ shapes, for better and for worse, a part of the beliefs they will hold about education. Subsequently, once they are part of the professional environment, these beliefs will be triggered when they encounter educational scenarios that are familiar to them, playing a role in the pedagogical decision-making and taking part in the political process of social reproduction of education (Ginsburg and Newman, 1985). This process can be described as a sort of “endogamy” that hampers educational system change (Bachler, 2017). Especially in situations of uncertainty, when the complexity of the classroom does not allow for technically planned practices, these representations or beliefs will play a predominant role in their practicing for even inexperienced teachers. Considering the above and the fact that the classroom is a place of permanent uncertainty and dynamism (Doyle, 1983; Torres, 1998) these representations can be understood as a key factor for the success of educational processes. Beliefs may be considered even more important than conveying expert knowledge (Tillema, 2000).

Despite the relevance of these beliefs, they are not often considered as a specific topic of work during ITT and therefore, they do not change during this process (Wideen et al., 1998). Richardson (2003) points out that the process of making held beliefs explicit is critical. On the contrary, it seems to be tacitly assumed that the process of becoming a teacher is one of an exclusively explicit nature that consists of “filling” student teachers with new knowledge as if they were empty jars, devoid of previous educational background. It is a perspective that recalls behaviorism and its conception of the mind as a clean slate since it comprehends teacher training as an additive process, which overlooks the person, his or her history and the relation he or she has with the environment (Feiman-Nemser and Buchmann, 1989). However, it would be desirable for ITT to consider an analysis and reworking of the meanings associated with the school experience of prospective teachers with the object of “releasing” them from the conditioning of their prior educational background. The first step for this change is to make explicit the educational conceptions that student teachers bring with them to ITT. Otherwise, the preservice teachers will incline in favor to their conceptions over the new knowledge (Egloff and Souvignier, 2020). Subsequently, there will be an interaction between these conceptions and the learning contents that shape the professional training. As Britzman (1991) points out, it is through a dialog between past and present voices that future teachers will be able to find their own voice as educators. In the words of Pozo (2008), this process consists of representatively rewriting these beliefs, thus constructing new meanings about education. The aim will be to bring to consciousness these experiential and intuitive representations, to give them an explicit and symbolic format by passing them through the sieve of the most updated knowledge on each educational topic particularly. When this happens, the representations will be decoupled from the educational situation that gave rise to them, thus transforming them into real theoretical knowledge, which can be applicable to different types of pedagogical contexts (Pozo, 2008). However, the process of changing the conceptions that student teachers bring to training is an arduous one and, contrary to the common sense associated with this topic, it does not consist solely and primarily of presenting logical arguments (Richardson, 2003). On the contrary, the presentation of logical arguments as the only strategy of intervention for changing conceptions may occasionally result in a sense of threat to the personal identity of students, a threat from which they will seek to defend themselves by weakening the strength of the arguments. In the words of Frijda and Mesquita (2001), “beliefs renders evidence powerless” (p. 45). Probably, this situation stems from the most fundamental fact that the ontology of implicit beliefs is ultimately emotional and not exclusively symbolic. Consequently, as stated Egloff and Souvignier (2020), changing beliefs during the ITT is a process in which emotions play a key role. Considering all the above, it can be understood that the process of changing the conceptions held by student teachers during the ITT involves a delicate balance between fostering affective states that are consistent with the proposed goals of change and, on the other side, accompanying the process through reflections that guide in a certain direction, also giving greater depth and control to the changes that are achieved (Smith, 2005). This requires that TEs have emotional and cognitive skills that can be brought into play during the process of teaching and learning. Among the emotional skills, it is desirable, for example, to be able to propitiate an environment of trust that is non-threatening to students in order to facilitate the expression of their points of views as freely as possible, given the significance that this has for learning (Coll et al., 2001). In the same vein, it seems convenient that teachers are able to read the non-verbal language of their students during the training process so as to modulate their own behavior according to this information. On the other hand, from a cognitive view, it is necessary that TEs develop explanations that make sense in the light of the perspectives held by the learners or, in other words, that they are able to build bridges between the new and the old and therefore avoid a “collision” between the two domains that would mean a rejection of the new information by the learners. Otherwise, in case of no adequate mediation of this process, student teachers are likely to remain tied to their earlier school experience and pedagogical action becomes, outdated due to its attachment to past experiences that lead it to lose the freshness and spontaneity of a conscious practice connected with the present (Naranjo, 2016).

### The conceptions and the role of teacher educators

Of course, the process of change we are aiming to occur, requires TEs who are aligned with the knowledge and skills to be achieved during ITT. Among the most important factors that influence the quality of higher education are the conceptions that teachers have about teaching. These conceptions influence teaching action and have a direct impact on students’ own academic performance and learning outcomes (Grácio et al., 2023). This is a difficult point to achieve, since among the professionals in the teams that train future teachers there are people with different specialties and experiences, which presuppose the existence of different types of educational conceptions (Alvarado, 2006). On the other side, we are talking not only about the need for the TEs to know the topics on which it is sought to train future teachers, but also to believe in them in an implicit, emotional, and deeper sense. Only when this happens is it feasible for TEs to implement pedagogical practices in their own educational space that are coherent with this focus and thus impact on the conceptions of their student teachers (Thomson and Palermo, 2014, p. 59). Otherwise, even if they are “updated “with the most current theories and techniques on a particular educational subject, their own practice as trainers is likely to “betray” them and lead them to implicitly communicate messages that are contradictory to what is explicitly and verbally stated (Riquelme, 2000; Watzlawick et al., 2002; Ng et al., 2010; Kelchtermans and Deketelaere, 2016). We do not have to forget that every educational process, even those at a higher level such as doctoral studies, always and inevitably involves implicit aspects that are not officially acknowledged in the curriculum (Beard et al., 2007; López-Goñi and Goñi-Zabala, 2012; Cotterall, 2013). Among these aspects, the non-verbal communication that teachers express during their pedagogical practice constitutes a central element of the educational process since it participates, most of the time involuntarily and unconsciously, in the shaping of the meaning attributed to what is expressed. This is due to the emotional character of that part of the communication, which acts as a kind of comment or meta-communication with respect to what is said verbally (Konar and Chakraborty, 2015) thus implicitly suggesting the way in which the message should be understood (Dimberg et al., 2000).

### The emotional education of student teachers

As we have affirmed, the topics examined in the previous section correspond to aspects that can occur in any teaching and learning process, regardless of the level of education involved. In school education as well as in undergraduate and postgraduate professional training processes, the non-verbal and emotional language associated with teachers’ implicit beliefs will always be a central dimension of everything that happens in the classroom. Certainly, the same happens during initial teacher training and, especially, when the content to be taught refers precisely to the role that emotions play in educational processes. We refer to a topic which is beginning to be considered with increasing depth within the ITT (Kelchtermans and Deketelaere, 2016; Bächler et al., 2020; Dernikos et al., 2020; Masse-Lamarche et al., 2022) due to the emotional turn that education is experiencing worldwide (Anzelin et al., 2020; Costa Rodriguez et al., 2021). In addition, because of the greater awareness of the role of emotions in educational processes after the pandemic generated by COVID-19 (Granda Granda and Granda Carrión, 2021) it seems more important than ever to promote among future teachers a renewed view on this topic that is aligned with current scientific knowledge on emotions. In this respect, an element that seems essential to consider is the recognition of the close interweaving of cognitive and affective processes (Gu et al., 2012; Bächler et al., 2018; Dejene, 2020; Bächler and Salas, 2021) and, therefore, the immanent presence of emotions in every educational process. If we want to develop an education that recovers its social and human character by abandoning the Taylorist, technicist, and standardizing visions that have caused so much damage to our education, we need a new generation of teachers who believe that emotions are at the core of every teaching and learning process and who develop pedagogical practices that break with the endemic emotional-cognitive dualism in our society and school systems (Searle, 1996; Claxton, 2005). This change implies promoting the idea that emotions are not only relevant when considering learning attitudes or learning about how to develop harmonious social relations at school, as seems to be the predominant point of view among our educators according to some studies (Hargreaves, 1998). On the contrary, it is a matter of understanding that even learning with a high symbolic content, such as mathematics, is based on the characteristics of our bodily and sensory structure, and therefore has an affective dimension associated with it (Gill and Hardin, 2015). On the other hand, a very important aspect of this paradigm shift implies that future teachers modify that idea so anchored in the common sense of our society that divides affection into positive and negative, understanding that this corresponds to an ontology lacking scientific support (Cabanas Díaz and González-Lamas, 2021) that has been encouraged by economic and political ideologies that conveniently seek to promote only one type of affectivity (Davies, 2016). This conceptual change also requires an awareness of the fact that educational culture is colonized by the emotional intelligence (Ciarrochi et al., 2000; Penalva, 2009), which has become the hegemonic paradigm when it comes to analyzing the role of affections in educational processes (Menéndez, 2018). Almost 30 years after the introduction of this model in our schools, this approach has ended up being an epistemological obstacle that makes it difficult to appreciate other ways of understanding the role of emotions in educational processes, reducing the role of affectivity in teaching, and learning to a problem consisting of learning how to control emotions. This is an ideology that is encouraged by another pseudo-science, the positive psychology, which confuses the subjective phenomenology of affective states with their adaptive nature, resulting in the idea that it is only possible to teach and learn from pleasant emotional states (of positive valence). The resulting coupling of these two viewpoints constitutes a perfect storm that distorts not only our ideas about emotions but also, more seriously, impacts on our conceptions of learning, reinforcing the idea that knowing is a representational and objective process where the subject and the object of the process are irretrievably separated (Atkinson and Claxton, 2010).

Teacher educators are the ones who possess the key to bring about this epistemological change, given the privileged position they have to intervene in the beliefs of future teachers, as it has been analyzed. Nevertheless, as we have exposed, for this to happen, it is first required that they themselves hold beliefs, about the role of emotions in educational processes, that are aligned with the most updated scientific knowledge on this area. In this context, knowing the conceptions or beliefs that they hold about this topic is the starting point for the whole process of change that we have been describing in the previous sections of this paper. However, paradoxically, since the labor of TEs is a recognized fact in education, the empirical research about professional living conditions of TEs has not had a substantial development. As mentioned by Vanassche and Kelchtermans (2014) “empirical research focusing directly on the professional lives and needs of teacher educators—those who teach teachers—remains scarce” (p. 117). Furthermore, in this general context of lack of studies on TEs’ characteristics, research on the conceptions that they hold has not mostly touched on emotional issues. On the contrary, these have focused preferably on cognitive aspects, such as the conceptions about teaching and learning process (Pozo et al., 2006; Grácio et al., 2023), the conceptions about the assessment of educational processes (Monteiro et al., 2021) or the conceptions about the intelligence of the students (Dweck, 1999) among others.

However, in recent years, the specialized literature has begun to highlight the need to address emotions in the study of conceptions:

We can no longer allow ourselves to ignore emotion when discussing teachers’ beliefs about learning […]; therefore, we suggest that research on teaching include an examination of the teachers’ emotions, alongside their beliefs, instead of separating these two fields of research, as is currently done in educational research (Gill and Hardin, 2015, p. 240).

To make progress in this knowledge, we need a model that enables us to identify different points of view on this field and, ideally, to be able to make comparisons with the perspectives held by other educational agents, such as student teachers and teachers in the school system. We discuss this issue in the next section.

### Conceptions of the role of emotions in educational processes

During the last decade, we have been engaged in the task of finding out what conceptions or beliefs teachers hold about the role of emotions in teaching and learning processes. This is not a study of conceptions about emotions (Tamir et al., 2007) or beliefs about learning as a cognitive process (Pozo et al., 2006). Our aim during this time has been to understand the conceptions that educators maintain over the way in which emotions participate in teaching and learning, on the understanding that, in the brain and the human mind, there is a close interweaving between emotional and cognitive processes (Duncan and Barrett, 2007; Pessoa, 2013). With this in mind, we started by identifying the viewpoints that might exist among teachers regarding this theme through a qualitative study (Bächler and Pozo, 2016). This research allowed us to identify different beliefs that teachers hold about the role of emotions in educational processes, which were named “behavioral reductionism” (hereafter referred to as BR); “influence of emotions on cognition” (hereafter referred to as IEC) and “emotional-cognitive integration” (hereafter referred to as ECI).^2^ Regarding the first conception, BR, this is a view that does not differentiate between emotions as subjective and private mental states and their associated behaviors. For this reason, when teachers who hold this perspective refer to affections, they use behavioral terms, pointing out, for example, that to be sad is to cry, as well as to be happy is to laugh. The second conception, IEC, refers to an approach, which assumes that all learning processes, including those that involve subjects with a high symbolic level dimension such as mathematics or history, are influenced by emotional states that students experience when they learn. This influence, however, is conceived in a simplified way based on the valence of emotions. From this approach, it is assumed without further hesitation that any emotion, of a pleasant nature or positive valence, is always a context that facilitates learning and, on the contrary, any unpleasant or negative affective state is, unequivocally, conceived as an obstacle to learning. This point of view is supported by an understanding of learning as a strictly cognitive process in the representational sense of the term (Fodor, 2001) and, therefore, it does not involve the participation of emotional aspects. Finally, the third conception, ECI, corresponds to a point of view, which, unlike all the previous ones, considers learning as an intrinsically emotional process. In this case, it is a perspective that understands that learning starts from an experience in the affective sense of the term. In this way, knowledge is conceived as the result of a reformatting process of sensations, emotions, and other affective and qualitative states, which constitute the starting point of knowing (Bachler, 2019). From this point of view, there is no ontological separation between emotional and cognitive states, in a line that is consistent with some neuroscientific perspectives that refer to a deep intertwining between these two types of phenomena in the brain (Damasio, 2001; Duncan and Barrett, 2007; Pessoa, 2013). On the other hand, given in this point of view that emotions are a central element of learning, the valence of affections is not a determining aspect of this process, because it is considered that both, pleasant emotions (of positive valence) as well as unpleasant states (of negative valence), can be a starting point to learn. Once the main viewpoints held by educational agents on the role of emotions in educational processes were identified and described, we carried out a second study aimed at finding out how these conceptions were distributed among a large and diverse sample of primary school teachers, from different universities, and with different sociodemographic characteristics (Bächler and Salas, 2021). To achieve this goal, we designed a questionnaire of dilemmas, a type of instrument that had already been used successfully in previous studies on conceptions. This research showed us that the influence of emotions on cognition (IEC) was the most widely accepted viewpoint among teachers, at the same time, there was a strong rejection of behavioral reductionism (BR), and a relatively neutral position toward emotional-cognitive integration (ECI). Subsequently, through a more limited study at a university, we investigated how these conceptions were reflected by first-year students of primary education (Bächler and Salas, 2021). In contrast to the case of practicing teachers, this time we found that the preferred conception by students was BR, followed by IEC, accompanied by a significant rejection of ECI.

Even though the two studies are independent from each other, the differences in the distribution of the conceptions give the opportunity to reflect on how teacher education could be a variable that substantially modifies these conceptions. Additionally, as we have previously discussed, it is also likely that the actions of TEs become a key factor in this change. For this reason, among others, the following study was designed with the aim of finding out what conceptions about the role of emotions in teaching and learning processes prevail in teacher educators.

## Materials and methods

The aim of this study was to assess the conceptions of teacher educators about the role of emotions in the teaching and learning process. The study uses an exploratory and quantitative approach with a descriptive cross-sectional design (Johnson et al., 2007) in order to characterize the conceptions of teachers and identify the profiles of teachers’ conceptions on the role of emotions in initial teacher training.

### Specific objectives

1. To determine which conceptions are preferred and which are rejected by TE.
2. To analyze and describe the differences between the conceptions held by teachers according to the type of learning content (verbal or attitudinal) to which emotions are associated.
3. To identify and describe conceptions profiles about the relations between emotions and the teaching-learning process.
4. To analyze and describe differences between the identified profiles according to the type of response they express toward emotions of negative and positive valence.

### Participants

The design of the sample was non-probabilistic by convenience, and it comprised 68 teachers who are teacher educators of primary education pedagogy from four universities in Chile, with an average age of 50 years and an average professional experience as TEs of 15.9 years. The inclusion criteria considered were:

- Teachers with an academic career in primary education.
- Teachers from universities belonging to the Council of Rectors of Chilean Universities.
- Classroom teachers.
- Teachers with at least half-time teaching experience in universities.

At the same time, teachers who hold head positions in teaching careers were considered as an exclusion criterion.

Table 1 presents some other characteristics of the sample.

**Table 1:** Sample characteristics.

Initial training	Disciplinary subjects	Pedagogical developments subjects	
Number of teachers	32	36	
Postgraduate training	Post title	Master	Doctorate
Number of teachers	9	40	19
Gender	Female	Male	
Number of teachers	35	33	

### Instrument

To assess conceptions, the study used a questionnaire of dilemmas, a type of instrument that had already been successfully applied in different conception studies (Vilanova et al., 2007; López et al., 2010). In this case, given that what was assessed were conceptions about the role of emotions in teaching and learning processes, we used an instrument that had already been considered for these purposes with a sample of primary school teachers (Bächler Silva and Pozo, 2020) and another with student teachers (Bächler and Salas, 2021). The questionnaire consists of 12 dilemmas that present educational situations where emotions are involved. For each dilemma, three response options are offered, each associated with one of the following conceptions about the connections between emotions and the teaching-learning processes previously analyzed: behavioral reductionism (BR), Influence of emotions on cognition (IEC), and Emotional-cognitive integration (ECI). Each participant was asked to choose the option they liked the most and the option they liked the least. This ranking allowed the responses to be tabulated by assigning a score of +1 for the preferred option and − 1 for the rejected option; the option left blank was scored as zero. On the other side, the instrument included two evaluation scales that enabled us to assess the existence of differences in conceptions according to the learning content in question. The first, consisting of four dilemmas, was called “students’ emotions and their relation to learning attitudes.” The second, consisting of eight dilemmas, was called “students’ emotions and their relation to the learning of verbal contents.” Each scale consisted of an equal number of dilemmas with emotions of positive and negative valence, which made it possible to compare if there were differences in conceptions according to the phenomenological “tone” of the emotions involved. In addition, to gain a deeper understanding of some features of the conception ECI when learning verbal content, two different types of items with emotions of positive valence were considered in the second scale. The first one proposed a situation under which such emotions could become an obstacle to learning, which implied that the teacher adopting an ECI conception should help the learner to realize the “risks” involved in feeling good in this scenario. Since, in this case, the option associated with the ECI conception involved decreasing the intensity of an emotion of positive valence; the maneuver was called “emotional inhibition for knowledge.” The following item is an example of this type of situation with the ECI response option highlighted in bold.

Maria is a student who is on risk of not achieving the minimum mark to pass English, because of not studying hard enough. However, in the assessment of this week, Maria has improved her grade slightly, so she is now happy and confident about her chances of passing the subject. In view of this situation, the teacher in charge:

Response option ECI (Emotional inhibition for learning): Increase the level of demand on Maria to help her realize that her confidence can play tricks on her.

The response option associated with the conception ECI consists, in this case, of an action carried out by the teacher that aims to modify the affection of the student. The idea is to “take” the student from a state of high intensity of positive valence to a state of lower intensity or even unpleasantness. The purpose is to facilitate an awareness of the “cognitive risks” associated with confidence along the same line of some authors who warn about a loss of attention to details when experiencing high intensity of pleasant emotions (Stein and Jewett, 1986; Anaya-Durand and Anaya-Huertas, 2010).

The other type of item with emotions of positive valence, that was included in the second scale of the questionnaire, considered situations in which teachers who adopted a conception of emotional-cognitive integration should take advantage of the pleasant affective state of students as a platform to deepen their learning. This modality could be implemented by increasing the level of complexity of the content or by extending its domain to other associated aspects. In both cases, it was a matter of using positive affect as a “trigger” to make the process more complex. Due to the above, in this case we call the response option associated with the conception of ECI “Deepening learning through emotions “. The following item is an example of this context:

The students are in science class working in the laboratory. During the session, the teacher notices that some students are restless due to their enthusiasm for experimenting with chemical transformations of matter. In response to this situation, the teacher decides:

Response option ECI (Deepening learning through emotions): Use the enthusiasm to deal with some aspects of science experimentation despite taking some extra time from other content development.

### Data analysis procedures

The work considered different analysis procedures. Previously, it was carried out an analysis using Kolmogorov–Smirnov Z-test to assess whether the variables considered in the study were normally distributed or not and, consequently, to determine the most appropriate type of statistic (parametric or non-parametric) for each analysis. Then, the following steps were taken.

To obtain an overall view of the acceptance or rejection of the conceptions expressed by the participants, the average responses to the entire questionnaire were calculated. Subsequently, we used a Wilcoxon test to determine if the differences between the obtained averages for each conception were significant or not. Secondly, to find out if the degrees of acceptance or rejection for each conception varied according to the different learning contents, the procedure described above was repeated, this time comparing the averages expressed for each conception on each of the two scales considered by the instrument using U Mann–Whitney test. Thirdly, we applied a multivariate technique, particularly, non-hierarchical cluster analysis, to identify the existence of groups of participants with differentiated responses that could be interpreted as conception profiles. Finally, seeking a deeper understanding of the differences between the more and less complex profiles, two specific analyses were carried out. The first was a comparison of responses between profiles according to the valence (positive or negative) of the emotions involved in the dilemmas. The second was a contrast of the degrees of acceptance or rejection expressed for each of the two specific maneuvers described above in the case of the ECI conception (deepening learning through emotions or emotional inhibition for learning). In the last two cases, the statistical test used was U Mann–Whitney.

## Results

The analysis using Kolmogorov–Smirnov Z-test shows an atypical behavior in the sample (*p* > 0.05). For this reason, most of the results presented below were obtained using non-parametric statistics. At the same time, we applied multivariate statistics.

### Results associated with specific objective 1: which conceptions teachers prefer and which they reject

**Figure 1:**
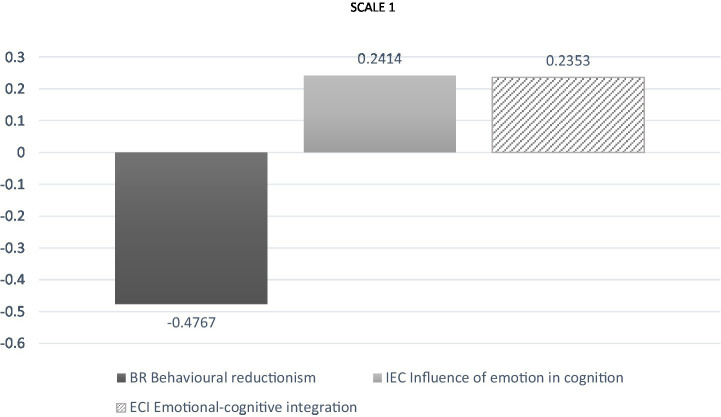
Acceptance average and rejection of each conception in the full questionnaire.

The results of this analysis are presented in Figure 1.

When considering the overall results obtained from the implementation of the questionnaire, without separating the responses per scale, the comparison shows that the average number of responses in relation to the BR conception is different from the average number expressed for IEC and for ECI (*p* = 0.001). However, no differences are detected for the comparison between IEC and ECI. The above allows us to affirm, firstly, the “behavioral reductionism” conception is the most rejected of all those evaluated. However, the conceptions “influence of emotions on cognition” and “emotional-cognitive integration” share the first place of preferences among the participants in the study (*p* = 0.001).

In the following subsection, we will go deeper into the analysis of the previous trends through the results referring to the preferred and rejected options by the participants, this time, in the different scales of the questionnaire.

### Results associated to the specific objective 2: analysis of conceptions according to the type of learning content (verbal or attitudinal) to which emotions are related

**Figure 2:**
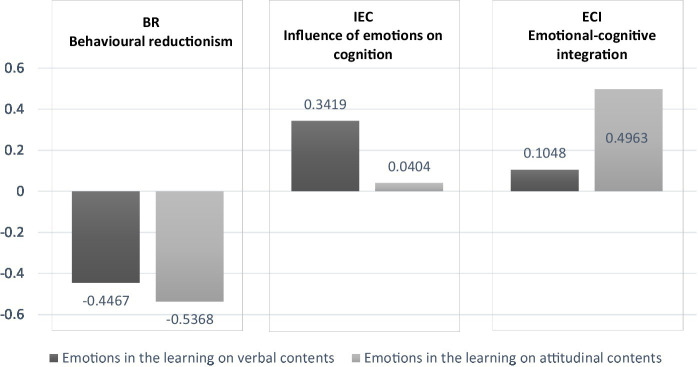
Conceptions about the role of emotions in the learning of verbal or attitudinal contents.

The results of this analysis are presented in Figure 2.

The analysis of the data shows a greater rejection of the behavioral reductionism conception when considering the learning of attitudes vs the learning of verbal content (*p* = 0.000).

In contrast, when considering the learning of verbal contents, there is a significantly higher acceptance for the IEC position, influence of emotions on cognition, compared to the level of acceptance reached for this option when considering the learning of attitudinal contents (*p* = 0.001). The above data show a tendency to consider emotions like a context of learning when it comes to learning verbal contents, a situation that is not appreciated when it comes to learning attitudinal contents.

At the same time, there are significant differences between the averages obtained for the ECI option, emotional-cognitive integration, when comparing the responses that are produced taking into account the learning of attitudinal contents vs. the learning of verbal contents. In the first case, there is a tendency toward acceptance of this option, while in the second case, the tendency is toward rejection (*p* = 0.000). The participants tend to consider an integration of emotions and cognition when learning attitudes, a conception that is rejected when considering the learning of verbal contents.

In summary, there is a tendency to acknowledge the role of emotions as the center of the learning process, (ECI conception, “emotional-cognitive integration”), when considering the learning of attitudes, rejecting the possibility of reducing affect to its associated behaviors (BR conception). On the other hand, when considering the learning of verbal content, the tendency is toward the consideration of emotions as the context of this process, their role being valued in terms of the valence of the affections (IEC conception, “Influence of emotions on cognition”).

### Results associated to the specific objective 3: identification of conception profiles

As previously indicated, the third objective of this study was to explore if all the information gathered in the preceding phases, was somehow organized into different profiles of conceptions about the role of emotions in learning processes.

In response to this point, multivariate statistics were accomplished, specifically, a series of cluster analyses using K means based on the results obtained for Scale 2, “Emotions of students and their relationship with the learning of verbal content.” The analysis of this scale was carried out understanding that this scale, being related to verbal learning, best represented the main goals and the most common type of activities in the school system, over and above any qualms that might be made about these aspects.

Regarding the results, different possibilities were considered in view of the number of clusters to be proposed, finally opting for a classification into three groups, which were interpreted in terms of conception profiles. The decision of considering three clusters was due to the fact that this number not only coincided with the conceptions addressed in the study, but also allowed for a better examination of differences regarding the degrees of rejection and preference for the conceptions. Apart from this, once the clusters to be considered had been determined and the general characteristics of each one had been described, a new analysis was carried out to compare, in a specific way, the two largest and most complex of them. For this purpose, first, the modes of response of each cluster toward the dilemmas of negative valence were analyzed. Subsequently, it was made an examination of the way of dealing with the two kinds of response associated to the ECI conception in items of positive valence emotions (emotional inhibition for learning and deepening learning through emotions).

Details of the results obtained for each of the analyses mentioned above are presented below.

### K-means cluster analysis considering three groups

The K-means clustering process allowed the grouping of participants within three clearly differentiated profiles, as can be seen in Table 2, with the first-choice tendency highlighted in darker gray, and the second one in a softer tone.

**Table 2:** Clusters resulting from the classification with the responses on Scale 2.

	Cluster 1 (16 cases, 23.52%)	Cluster 2 (21 cases, 30.88%)	Cluster 3 (31 cases, 45.59%)
Behavioral reductionism average	−0.06	−0.56	−0.57
Influence of emotions on cognition average	0.36	0.52	0.21
Emotional-cognitive integration average	−0.30	0.04	0.35
First-choice tendency			
Second-choice tendency			

The main characteristics of each cluster, interpreted as conception profiles, are presented below.

a. Profile 1: “Influence of emotions on educational processes: Emotions as a context for learning processes” (16 cases).

It corresponds to a profile that mostly follows the conception “influence of emotions on cognition” with a neutral attitude toward “behavioral reductionism” and shows a clear rejection of “emotional-cognitive integration.” This is the most basic conglomerate emerged from the analysis and corresponds to a profile that tends, on some occasions, to ignore the mental status of emotions by reducing affect to its associated behaviors (BR conception). However, the position, that is most frequently adopted by participants who adhere to this profile, would be to consider emotions as the context of learning processes (IEC conception), although this implies a recognition of the role of affections on cognitive processes, it is a perspective that is grounded in a cognitive-emotional dualism. Furthermore, from this point of view, the role of the valence of emotions on learning processes is considered in a simple way, since it is assumed that any pleasant emotion is favorable for learning, while, on the contrary, unpleasant “tone” affects are unequivocally conceived as obstacles to this process.

b. Profile 2: “Transition toward emotional-cognitive integration” (21 cases).

This is a profile that shares with the previous one, a major preference for the “Influence of emotions on cognition” conception. However, in this case, unlike the first one, there is a position of firm rejection of “behavioral reductionism” accompanied, moreover, by a neutral position toward “emotional-cognitive integration.” The above implies that, in some situations, teachers who adhere to the second profile consider learning as an affective process, without making ontological distinctions between emotion and cognition (ECI conception) and they also value the role of affections in the learning processes beyond their valence. Given the above characteristics, this profile can be considered as a stage of probable transition toward the more complex profile described below.

c. Profile 3: “Emotional-cognitive integration: emotions as the core of learning processes” (31 cases).

The third profile exhibits a strong preference for the most complex conception of all those considered in this study (ECI conception), followed by an appreciation of emotions as a context of learning processes (IEC conception) and a clear rejection of the BR conception. The foregoing information can be interpreted as affirming that TEs adhering to this profile maintain a perspective on learning as an emotional process, thus diluting the cognitive-emotional dualism that features the previous profiles. From this point of view, it is understood that to feel is to learn and, therefore, the role of the valence of emotions involved in cognitive processes leaves the scene, giving rise to the idea that any emotional state, regardless of its subjective phenomenology, can be the beginning of a learning process.

A characteristic that the different profiles identified have in common is that they all present, on one hand, a main point of view or tendency to value the role of emotions on learning processes, accompanied, on the other hand, by a secondary perspective. This means that the first profile is predominantly under the IEC conception, but secondarily under the BR conception, while the second profile is predominantly under the IEC conception, but with a preference for the ECI conception in some cases. And the third profile reverses the trend of the previous profile with a stronger preference for the ECI perspective followed by a less robust appreciation of the IEC conception. This characteristic of the profiles is interesting, especially if we assume the conceptions studied as a progression from simpler to more complex positions, as actually proposed in different studies (Pozo et al., 2006; Scheuer et al., 2010; Bächler and Pozo, 2016). With this logic in mind, it is possible that the differences between the profiles can be explained by some of the characteristics of the educational situations, which were part of the dilemmas in the evaluation questionnaire. In other words, it is possible that certain features of the dilemmas make it easier or more difficult for the participants in the study to adopt more complex perspectives to assess the role of affections in learning processes. In relation to this point, it seems especially relevant to know what kind of characteristics of the dilemmatic situations could indicate the turning point that facilitates a shift from preferring IEC followed by ECI (profile 2), toward one in which this relationship is reversed, transforming the conception of emotional-cognitive integration into the predominant point of view (profile 3). In the following section, two possible characteristics of dilemmatic situations that could be related to this point are analyzed.

### Analysis of the differences of response between profile 1 and profile 2 toward ECI conception

Considering the methodological design of our study and the characteristics of the instrument used, there are two aspects that can be considered to examine the issue described above. The first consists of an investigation of the possible differences in response to the ECI conception that might exist between profiles 2 and 3 according to the type of valence of the emotions involved in each dilemmatic situation. The second corresponds to an evaluation of possible differences with respect to the way of weighting the more complex conception (ECI) when the dilemma offers two different response alternatives associated with this conception, namely, emotional inhibition for learning and deepening learning through emotions.

Regarding the first, when comparing response tendencies to the ECI preference in items with emotions of negative valence, vs. those consisting of situations where emotions of positive valence are involved, we observe that for both profiles it is more difficult to adopt the ECI conception when the dilemmas contain emotions of positive valence (*p* = 0.002). Nevertheless, when comparing the average number of ECI responses on the positive valence items for each profile, it is observed that profile 3 expresses a less intense rejection of the ECI position compared to the degree of rejection that profile 2 expresses on these items (*p* = 0.001). For this reason, we decided to “focus” on these items in order to find out which of the two specific types considered in this study for the ECI option, deepening learning through emotions or emotional inhibition for learning, represented a greater problem when choosing this option in each profile. The results of the analysis show that, for both profiles, there is a lower probability of choosing the ECI conception when the ECI response option is emotional inhibition for learning (*p* = 0.001). However, when comparing the degrees of rejection of this option in each profile, we find that it is higher in profile 2. In other words, those who adhere to profile 3 have less difficulty in accepting the maneuver called “emotional inhibition for learning” when choosing the ECI conception.

## Discussion and conclusion

In the following, some of the reflections that emerge from all the work carried out are presented. This section has been organized into three parts. The first refers to general questions linked to the importance of the topic studied and the possibilities for the development of the line of research proposed by the study. The second part presents reflections directly related to the results obtained in order to facilitate their interpretation. Finally, it is offered an appreciation of the limitations that the work carried out presents.

## General reflections

As analyzed in the foundations of this study, the previous literature about conceptions of TEs is scarce, therefore, it can be stated that this study contributes to the comprehension of a topic that despite its relevance, has not been examined in sufficient detail. This situation is further enhanced by the fact that it deals with conceptions of the role of emotions in educational processes, a topic that is becoming increasingly significant in education. Simultaneously, it is necessary to highlight the importance of having studied the conceptions that TEs have about emotions and their role in educational processes based on a perspective that is different from the one promoted by the educational establishment of emotional intelligence and positive psychology (Menéndez, 2018), given the dualistic and reductionist characteristics that these approaches propitiate as we explained. In the same way as occurs with the change in educational systems, which in part involves a modification of the conceptions of the different educational agents, it seems pertinent to reflect on the perspectives held by researchers and how these can contribute to strengthening the status quo of education. Current studies on the evolution of scientific knowledge over the last decades point out that science is, in general, becoming decreasingly disruptive (Park et al., 2023), a situation to which educational research does not seem to be oblivious. In this context, although we are currently experiencing an emotional turn in education (Dernikos et al., 2020), a trend that, *a priori*, can be assessed as positive, since it breaks the historical denial of emotions in our culture, no less true, as analyzed, is the fact that this turn is colonized by approaches lacking the necessary scientific substantiation required by educational practices based on evidence. In this scenario, if we wish to move in a new direction, we need research that, along similar lines, addresses the emotional dimension of teaching and learning processes without the biases of emotional intelligence and positive psychology.

On the other hand, along with highlighting the importance of the study carried out, it is also necessary to point out that further research on the characteristics of TEs in general is required, beyond the study of the conceptions they hold about the role of emotions in educational processes. How is it possible that a large amount of research is carried out with practicing teachers in the school education system and no equal or greater attention is devoted to understanding the characteristics of those who train these educational agents? Substantial improvement in the quality of future teachers may not be possible if we do not begin by first understanding, which aspects should be part of TEs training. In relation to this point, there are some studies that identify subjects that should be compulsory topics in training (Goodwin and Kosnik, 2013), but there are also others that affirm that there are no essential topics in this regard (Ping et al., 2018). The study of educational conceptions held by TEs is probably a good starting point for moving toward greater consensus on this issue.

Finally, it seems evident that a step forward in the development of the line of research that this paper proposes is the analysis of the eventual relationships between the conceptions held by TEs and the pedagogical practices that they implement in their classrooms. On this point, it is pertinent to wonder whether there is any association between the profiles of conceptions found and different affective skills in the TEs. Since the literature reviewed shows that logical argumentation is not sufficient to change the conceptions of student teachers, it is desirable that those TEs who hold more complex conceptions about the role of emotions in educational processes accompany their perspectives with emotional skills that allow for a greater formative impact among their students. Future studies should undertake research in this area.

## Reflections about the results

### Overall response trends

The work carried out has allowed us to obtain, first, an overall assessment of the conceptions preferred and rejected by TEs that participated in this study. On this point, it is strikingly positive that the trends found reflect, in first place, a clear rejection of the most basic of all the conceptions considered in the study, “behavioral reductionism,” as well as similar degrees of adherence to the more complex conceptions “influence of emotions on cognition” and “emotional-cognitive integration.” These results are heartening since, if we assume that the TEs are mainly responsible for eventual epistemological changes in future teachers, then it is necessary that they hold more complex positions than those brought by student teachers who, according to the only study done so far, show a significant adherence to behavioral reductionism for this group (Bächler and Salas, 2021). In this context, a minimum requirement for fostering changes is, perhaps, the recognition of emotions as private, subjective mental states that are distinct from behavior, as can be seen from the strong rejection of behavioral reductionism expressed by the sample of participants in this study. But, in addition, the adherence that the TEs expressed to the conception called “influence of emotions on cognition” shows that they conceive the emotions as states that play a role in learning processes and that, in some cases, given the assessment expressed about ECI, the TEs even consider learning as an emotional process. With regard to the latter, however, this is a fact that is more pronounced when attitudinal learning is taken into account. What are the reasons why it is assumed that, when learning attitudes, unlike what would happen when learning mathematics or history, emotions are considered to play a central role in this process? We elaborate some reflections on this issue in the following concluding section.

### Variation in conceptions when considering different types of learning

As was the case when we assessed this point with practicing primary school teachers (Bächler Silva and Pozo, 2020), TEs tend to adopt a more complex perspective on the role of emotions when thinking about attitudinal learning compared to the view that they hold when considering verbal learning. It seems to be assumed that learning attitudes, unlike learning about language or chemistry, is an intrinsically emotional process. This means that when thinking about changing attitudes, it is understood that this process is not solely accompanied or influenced by emotions, as seems to be the most popular understanding of learning verbal content. On the contrary, in the case of attitudes, the very process of learning would consist of doing something with that which is felt or experienced. Moreover, when TEs think about attitude learning, they consider that the valence of the emotions involved does not determine the quality of the outcome as it does, at least in some cases, when TEs consider verbal content learning. It is likely that behind these differences lies a remnant of the idea that learning verbal content is a strictly cognitive process in the most basic representational sense of the term. We refer to the conception of learning as reflection or copying, a belief that is affirmed in a naive realism, which holds that human beings have direct and objective access to reality (López-Goñi and Goñi-Zabala, 2012). New studies that consider the relationships between conceptions about learning and beliefs about the role that emotions play in this process should enhance our understanding of this issue. However, this is a perspective that would not be in the majority among TE, as shown by the results of the cluster analysis discussed below.

### Possibility of interpreting the identified clusters as a progression of viewpoints

As previously analyzed, the TE, unlike other educational agents such as student teachers or practicing teachers, show a higher degree of adherence to the more sophisticated conception considered in this study called “emotional-cognitive integration”(ECI). This characteristic is observed in the general response trends, but also in the fact that the largest cluster of all those identified in this study corresponds to one that has as a distinctive feature its preference for the ECI conception. Then, if clusters are assumed as part of a continuous progression from more dichotomous and dualistic positions to the adoption of more integrative views, it is relevant to know what would be the turning point that would allow reaching the more complex position. The question refers to the identification of barriers for the transition from one profile that is closer to the predominant emotional status quo in our culture to one that is more complex and aligned with current knowledge about the role of emotions in cognitive processes. The results obtained on this topic show that this point would be found within the consideration of positive valence emotions, especially when the necessary actions to be taken by teachers to progress in the learning process consist of a questioning of the positive mood that a student may have at a given moment. It is about recognizing those moments in which positive emotions can become an obstacle to learning, generally because they hamper a slow and reflective analysis of the learning content (Anaya-Durand and Anaya-Huertas, 2010). In this context, a maneuver that facilitates students’ awareness of the “risks” of feeling good becomes an action that expands learning possibilities. However, it seems that this kind of action implies a frontal clash with the prevailing emotional culture that claims that feeling good should be always privileged. On the other hand, we also know that our school system is burdened with a heavy history of suffering among students (Polizzi and Frick, 2023), which restricts the maneuvering possibilities of current teachers. Within this scenario, it may be the case that TEs avoid any action that could assimilate them to the figure of a vindictive or punitive teacher, just as some studies show that there is a relationship between their own experiences of suffering as a student in the school system and the conceptions they hold about the role of emotions in educational processes as a future teacher (Bächler and Salas, 2021). This is a dimension dependent on the characteristics of the education system that reinforces an emotionally limited way of acting. And this way of seeing things is also reinforced by the very psychological configuration of the human mind, which naturally tends toward the pursuit of pleasure and happiness as a healthier and more adaptive mode of functioning than which is associated with suffering and pain. Nevertheless, as it occurs with some people with regard to developmental processes, who over the years tend to relativise the value of positive emotions (López-Pérez et al., 2023), building conceptions about joy that are more focused on eudaimonic well-being and not so much on hedonic pleasure (López-Pérez et al., 2016) deeper degrees of wisdom that lead them to also question the idea of displeasure as an intrinsically negative state of mind, we should ask ourselves, what to do to move in that direction. This fact becomes even more relevant if we assume teaching as an intuitive process (Atkinson and Claxton, 2010), in other words, a type of practice that is not exclusively rational in the explicit sense of the term. We refer to the fact that pedagogical decision-making often depends on bodily signals or somatic markers (Damasio, 2001) that teachers experience during the process. If this is so, how to deny a whole range of experiences of negative valence that can be profoundly informative of what happens in the classroom. Similarly, thinking now about learning as an emotional process, it is worth wondering what is lost when we compulsively seek pleasure in learning in a superficial sense of the term. To what extent do we restrict the possibilities of knowledge acquisition when we discredit, for example, the discomfort caused by a point of view that is contrary to our own ideas? How much is lost when we deny the uneasiness involved in learning about an intrinsically sad process such as illness or deterioration?

It seems reasonable to ask teacher educators to go one step beyond this culturally established common sense, thus “opening” the minds of their students and future teachers to interrupt the educational endogamy that characterizes our school systems. As we have pointed out, the first step is to encourage the recognition of existing conceptions about the role of emotions in the learning processes, an aspect to which we have sought to contribute with this work. However, much more than that is required; in particular, it seems convenient to analyze how the conceptions held by TEs are related to different types of pedagogical practices during the training of future teachers. In this context, the first point to consider, refers to the ability of TEs to facilitate the process of making explicit the conceptions that student teachers hold. As was examined, given the implicit and emotional nature of these conceptions, making them explicit is a complex process since it requires the use of skills that are not only cognitive. In the same line, future studies should also investigate the existing relationships between the conceptions held by TEs and different ways of persuading students to change their conceptions, considering that changing them does not only or mainly involve the presentation of logical arguments. In relation to the above, it is important to investigate the educational processes and micro-processes that take place in teaching careers and that have the greatest impact on the change of students’ conceptions. We are referring to both strategies that have been designed to produce these changes, as well as to other types of processes that occur fortuitously or implicitly and that nevertheless play a role in modifying the beliefs that student teachers bring to the course. Future research will have to assume this task.

### Limitations of the study

Finally, to warn about a limitation that we observed in our study. We refer to the fact that, with the obtained data, we are not in a position to determine accurately what is, exactly, the emotional background from which the alternative responses to dilemmas are chosen. This situation is a problem if we assume, as we have done in this paper that the ultimate meaning of any communicative process results from the interaction between symbolic aspects associated with verbal language and emotional components linked to non-verbal language. This aspect is of crucial importance in the case of the response alternative associated with the concept of “emotional-cognitive integration,” which we have called “emotional inhibition for learning.” As we have stated, this is an option that would aim at facilitating an awareness in learners of possible “risks” that feeling good may imply for learning. It can be assumed that those who choose this alternative focus on possible improvements in the learning process of their students. However, as we well know with respect to the educational system, there are not many cases of teachers who carry out similar maneuvers, in the sense of modifying the pleasant mood of their students, for different and not always positive purposes. In this context, we cannot exclude that among the cases of teachers that we have included in profile 3 of our study, some teachers of this type have “slipped in.” For this reason, it seems important to carry out further qualitative studies in the future to investigate the emotional background of the different profiles.

## Data availability statement

The raw data supporting the conclusions of this article will be made available by the authors, without undue reservation.

## Ethics statement

The studies involving human participants were reviewed and approved by Comité de bioética de la Universidad de Playa Ancha. The patients/participants provided their written informed consent to participate in this study.

## Author contributions

RB contributed to the design, planning, and data collection process of the study. RB and PS-L contributed to the data analysis phase, discussion of results, drafting and writing of the final manuscript, as well as its final approval. CP contributed to the data collection process and operational aspects as a researcher in training. All authors contributed to the article and approved the submitted version.

## Funding

This article was supported by “*Agencia Nacional de Investigación y Desarrollo de Chile* (*ANID*),” through its “*FONDECYT Iniciación*” program, which financed the project 11220861, entitled: “*Evaluación de la formación emocional inicial docente de las carreras de pedagogía en educación básica de Chile*.”

## Conflict of interest

The authors declare that the research was conducted in the absence of any commercial or financial relationships that could be construed as a potential conflict of interest.

## Publisher’s note

All claims expressed in this article are solely those of the authors and do not necessarily represent those of their affiliated organizations, or those of the publisher, the editors and the reviewers. Any product that may be evaluated in this article, or claim that may be made by its manufacturer, is not guaranteed or endorsed by the publisher.

## Footnotes

^1^In this study we use the terms “conception” and “belief” as synonyms.

^2^The study identified four different perspectives; however, one of them was excluded later, for being considered that it could be understood as a specific scenery in new studies.
